# Genetic Characterization of Avian Paramyxovirus Isolated from Wild Waterfowl in Korea between 2015 and 2021

**DOI:** 10.3390/ani14050780

**Published:** 2024-03-01

**Authors:** Yea-Jin Lee, Jong-Yeol Park, Ke Shang, Jun-Feng Zhang, Yu-Ri Choi, Sang-Won Kim, Se-Yeoun Cha, Min Kang, Bai Wei, Hyung-Kwan Jang

**Affiliations:** 1 Department of Avian Diseases, College of Veterinary Medicine and Center for Avian Disease, Jeonbuk National University, Iksan 54596, Republic of Korea; lyj95923@naver.com (Y.-J.L.); jyp410@naver.com (J.-Y.P.); shangke0624@gmail.com (K.S.); jfzhang018@gmail.com (J.-F.Z.); 9cinderella7@naver.com (Y.-R.C.); sk970221@gmail.com (S.-W.K.); seyeouncha@jbnu.ac.kr (S.-Y.C.); vet.minkang@gmail.com (M.K.); 2 Bio Disease Control (BIOD) Co., Ltd., Iksan 54596, Republic of Korea

**Keywords:** avian paramyxovirus, Korea, wild waterfowls, phylogenetics, intercontinental transmission, APMV-1, APMV-13

## Abstract

**Simple Summary:**

Wild animals can be of veterinary and public health concern due to their potential to harbor pathogens and transmit them to humans and domestic animals. Avian paramyxovirus 1 (APMV-1) is capable of infecting a wide range of avian species, leading to a broad spectrum of clinical symptoms and posing a potential risk to public health. This study represents the investigation of avian paramyxoviruses (APMVs) in wild waterfowl from Korea. In this study, 13 isolates of APMV-1 and 1 isolate of APMV-13 were obtained and their genetic characteristics of fusion gene were analyzed. We identified the wild ducks and geese likely to be infected with APMV, and our data suggest a possible intercontinental transmission of APMVs by wild waterfowl. Moreover, our study provides the first evidence for the presence of class I of APMV-1 and APMV-13 in wild waterfowl that were sampled in Korea. This finding not only adds to our understanding of the diversity of APMVs but also underscores the importance of monitoring and addressing the epidemiology of these viruses in wildlife, particularly from a One Health perspective.

**Abstract:**

Avian paramyxoviruses (APMVs) are often carried by wild waterfowl, and the wild waterfowl may play an important role in the maintenance and spread of these viruses. In this study, we investigated APMVs in the population of migratory wild waterfowl from 2015 to 2021 in Korea and analyzed their genetic characteristics. Fourteen viruses were isolated and subsequently identified as APMV-1 (n = 13) and APMV-13 (n = 1). Phylogenetic analysis of the full fusion gene of 13 APMV-1 isolates showed that 10 APMV-1 isolates belonged to the class II sub-genotype I.2, which was epidemiologically linked to viruses from the Eurasian continent, and 3 viruses belonged to class I, which linked to viruses from the USA. The APMV-13 isolates from wild geese in this study were highly homology to the virus isolated from China. Sequence analysis of 14 isolates showed that all isolates had a typical lentogenic motif at the cleavage site. In summary, we identified the wild species likely to be infected with APMV and our data suggest possible intercontinental transmission of APMV by wild waterfowl. Our current study also provides the first evidence for the presence of class I of APMV-1 and APMV-13 in wild waterfowl surveyed in Korea.

## 1. Introduction

Avian paramyxoviruses (APMV) belong to the genus Avulavirus of the Paramyxoviridae family, and, to date, they have been found to cause diseases with varying clinical manifestations in more than 250 wild and domestic bird species [1]. There are currently three genera (Metaavulavirus, Orthoavulavirus, and Paraavulavirus) within Paramyxoviridae, including 21 described APMV viruses based on genetic sequencing as per recent classification of International Committee on Taxonomy of Viruses (Virus Taxonomy: 2020 Release). Additionally, an APMV virus, APMV-22, has been recently proposed [2]. All paramyxoviruses carry a single-stranded, negative-sense, and non-segmented RNA genome with a genome length of 13–17 kb. The virus genome encodes six non-overlapping structural proteins, including nucleocapsid (NP), phosphoprotein (P), matrix (M), fusion (F), hemagglutinin–neuraminidase (HN), and large polymerase (L) [3]. The existence of mono-/multi-basic amino acids at the F protein cleavage site is a key indicator of virulence, and based on the nature of the cleavage site, paramyxoviruses are classified into velogenic (highly pathogenic), mesogenic (intermediate pathogenic), or lentogenic (low pathogenic) strains [4,5]. Among the 22 viruses of APMV (the nomenclature used is not the official one but the traditional one, based on serotypes), APMV-1, also known as Newcastle disease virus (NDV), causes severe disease in chickens and incurs devastating economic losses for poultry industries [3,6]. Other APMVs have no significant economic impact on the poultry industry; however, some viruses of APMV, including APMV-2, APMV-4, APMV-5, APMV-7, and APMV-9, could cause experimental respiratory diseases and reproductive problems in chicken, turkeys, ducks, and ostriches [3]. The virus of APMV-13 was isolated from wild waterfowls in countries such as Japan, Ukraine, and Kazakhstan, and most recently from Chinese wild waterfowls, and was confirmed to be non-pathogenic to chickens [7,8,9,10].

Generally, waterfowls have been considered a natural reservoir for APMV and long-distance vectors for APMV dissemination to poultry. More importantly, domestic poultry-origin APMV strains isolated from ducks and chickens could also transmit to wild waterfowls, particularly for the wintering sites for wild waterfowls [11,12]. In addition, there is also evidence of APMV transmission from poultry to wild waterfowl species, particularly since wild waterfowl were reported to harbor a highly virulent strain [13,14,15]. South Korea is an important wintering site for wild migratory birds, such as waterfowl, found on the East Asia–Australian flyway [16]. More than 250 species of wild waterfowl pass through the Korean peninsula in the winter [17]. The ecosystem has shown wild waterfowls as an important medium for the dissemination of infectious diseases via their natural migration; accordingly, many reports have shown South Korea is an important site for surveillance of infectious diseases, including avian influenza virus (AIV), in wild waterfowls [18,19,20]. The potential for AIV and APMV-1 dispersal and transmission is high because many migratory waterfowl, such as wild ducks, wild geese, and swans, overwinter in Korea after migrating from Alaska, eastern Siberia, eastern Mongolia, and the Russian Far East. Furthermore, many APMV viruses (APMV-4, APMV-6, APMV-15, and APMV-21) have also been found in wild waterfowl species passing through Korea [21,22,23,24]. Consequently, continuous and systemic surveillance of APMV among wild waterfowls and domestic poultry is important for providing helpful information on the occurrence and dissemination of APMV. Here, we conducted an elaborate survey of APMV and isolated the APMV strains from migratory wild waterfowl from 2015 to 2021 in Korea.

## 2. Materials and Methods

### 2.1. Sample Collection and Preparation

In this study, 1536 pooled fecal samples (2015–2021) were obtained from an avian influenza virus national monitoring program. The samples were collected throughout the whole year including 223, 85, 534, and 694 samples in spring, summer, autumn, and winter, respectively (Table S2). The fecal samples were collected throughout Korea by the South Korea Animal and Plant Quarantine Agency (QIA) and the Ministry of Agriculture, Food and Rural Affairs (MAFRA) [25,26,27], including the areas of Chungbuk, Chungnam, Jeonbuk, Jeonnam, Gyeongbuk, Gyeongnam, Jeju, and Gyeonggi (Figure S1). All samples were placed in a 15 mL conical tube with sterile phosphate buffer saline (PBS) containing antibiotics (penicillin 10,000 units/mL, streptomycin 10,000 µg/mL, and amphotericin B 25 µg/mL). The samples were immediately transported to the Center for Avian Disease of the Jeonbuk National University and stored at −80 °C until further processing.

Ethical review and approval were not required for this work as fecal samples were collected from the environment and no wild birds were handled or euthanized.

### 2.2. Virus Isolation and Detection

Approximately 1.0 mL of each sample was filtered through a 0.22 μm syringe filter (Millipore Millex™, Billerica, MA, USA). Then, 0.2 mL of the filtrate was inoculated in 9–11-day-old specific-pathogen-free (SPF) embryonated chicken eggs for blind passage and incubated at 37 °C for 3–5 days, as per a previously described protocol [5]. The allantoic fluids were harvested and tested for hemagglutinin (HA) activity and subjected to F gene-based reverse transcription polymerase chain reaction (RT-PCR), as previously described [28]. The positive rate was calculated as positive rate = (Number of positive samples/Number of total samples) × 100.

### 2.3. Sequencing and Phylogenetic Analysis

Samples that were both HA- and RT-PCR positive were further characterized for the complete F gene by nucleotide sequencing; the F gene was amplified by RT-PCR as previously described [10,29]. DNA fragments of the expected size were purified using the QIAquick^®^ Gel Extraction kit (QIAGEN GmbH, Hilden, Germany), and purified DNA was cloned using a pGEM-T-Easy cloning kit (Promega, Madison, WI, USA). The purified amplicons were subjected to sequencing in an ABI PRISM 3100 DNA Sequencer (Applied Biosystems, Foster, CA, USA) using a BigDye Terminator v3.1 Cycle Sequencing Kit (Applied Biosystems, Foster, CA, USA). A Basic Local Alignment Search Tool (BLAST) search was performed for all obtained sequences against GenBank’s APMV sequence. Phylogenetic analysis of the complete F gene sequence was performed with the molecular evolutionary genetics analysis program MEGA X using a maximum likelihood analysis [30]. Statistical analysis of phylogenies was performed by bootstrap analysis with 1000 iterations.

### 2.4. Host Species Identification

Host species identification was performed for HA- and RT-PCR-positive feces samples using DNA barcoding technology, as previously described [31]. Briefly, host DNA was extracted from the fecal supernatant using the Accuprep fecal DNA extraction kit (Bioneer, Daejeon, Korea) according to the manufacturer’s instructions. PCR was performed using the primers Aves-F (5′–GCATGAGCAGGAATAGTTGG–3′) and Aves-R (5′–AAGATGTAGACTTCTGGGTG–3′). The amplified DNA products were verified by 1.5% agarose gel electrophoresis. Using the direct sequence method, the gel of the target band was cut and analyzed by SolGent (Daejeon, Korea). The mitochondrial cytochrome oxidase gene was compared to a sequencing database provided by Barcode of Life Data.

### 2.5. Accession Number

The complete F gene sequences of obtained APMV-1 and APMV-13 strains were submitted to the GenBank database and are available under the accession numbers MW464627-MW464636, MW492545, and MZ579651-MZ579653.

## 3. Results

### 3.1. Virus Detection and Identification

Large-scale monitoring of wild waterfowl, which was focused on APMV in this study, was conducted in 2015–2021 and resulted in 1536 samples; the sampling sites covered almost all migrating birds’ resting sites throughout Korea. From among the 1536 samples, 14 samples were APMV positive for HA, RT-PCR, and the isolation rate was 0.9% (Table S1). APMV isolation rates were 2.4%, 1.6%, 1.5%, and 6.7% in 2016, 2017, 2020, and 2021, respectively. No APMV was isolated from wild waterfowl in 2015, 2018, and 2019. APMV was isolated from wild waterfowl collected in the spring, autumn, and winter, with isolation rates of 0.4%, 1.9%, and 0.4%, respectively (Table S2). APMV was not isolated from wild waterfowl in the summer. All 14 APMV strains were isolated in birds belonging to the Anatidae family, which includes ducks and geese, with 10 strains isolated from ducks and 4 strains from geese (Table 1). Among these 14 strains, 6 APMV strains were isolated from mandarin duck, 2 strains each from mallard duck, greater white-fronted goose, and taiga bean goose, and one strain each from Indian spot-billed duck and Eurasian teal.

### 3.2. Genetic Characterization of AMPV-1

**Figure 1:**
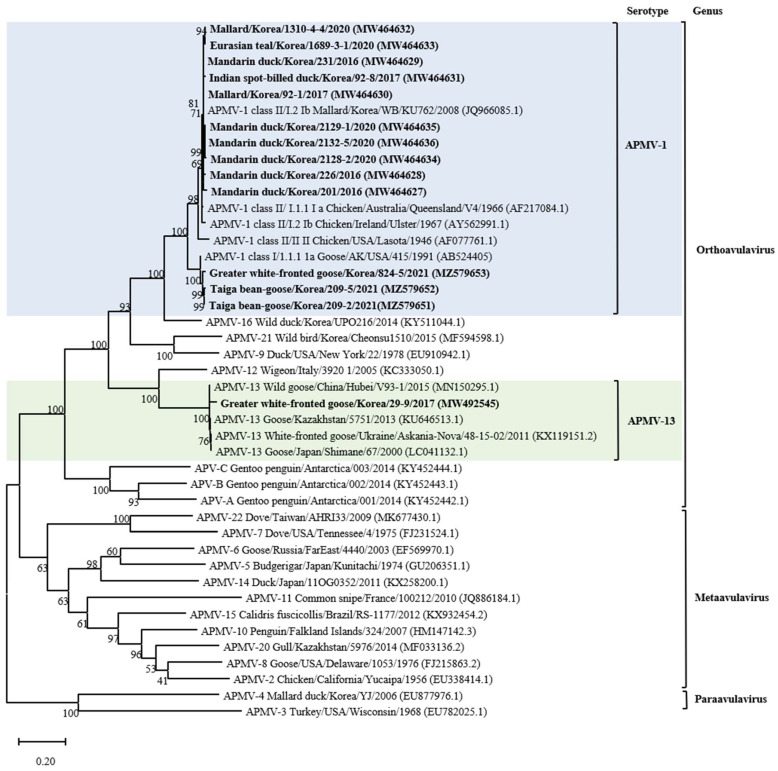
Maximum likelihood phylogenetic analysis based on the full-length amino acid sequence of the fusion gene of selected APMVs. Viruses described in this study are designated in bold. The new (decimal naming) and the former names (alpha-numerical) of genotype or sub-genotype in APMV-1 are provided for easier comparison. The viruses to which the newly isolated strain belongs are color highlighted for emphasis.

**Figure 2:**
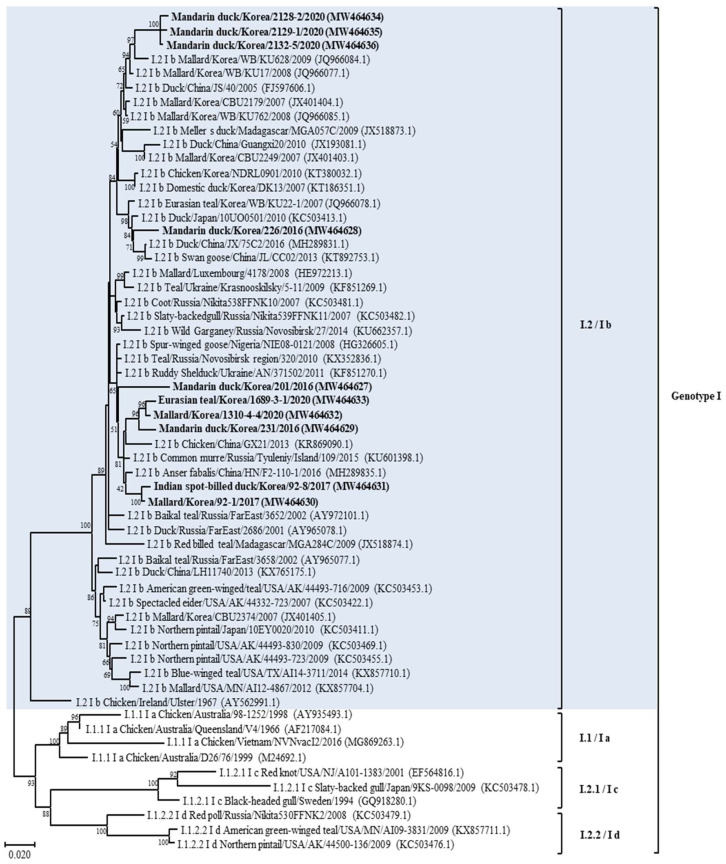
Phylogenetic analyses based on the full-length nucleotide sequence of the fusion gene of isolates representing APMV-1 class II genotype I isolates. Viruses described in this study are designated in bold. The new (decimal naming) and the former names (alpha-numerical) of genotype or sub-genotype in APMV-1 are provided for easier comparison. The genotype to which the newly isolated strain belongs has been color-highlighted for emphasis.

According to the phylogenetic analysis of the F gene, it was established that 13 isolates belonged to APMV-1, as shown in Table 1 and Figure 1 and Figure S2. The full fusion protein gene (1662 bp) sequences of the 10 class II isolates were most closely related to those previously identified as sub-genotype I.2 (former sub-genotype Ib) illustrated in the phylogenetic tree constructed using the maximum likelihood analysis (Figure 2 and Figure S3). Isolates 2128-2, 2129-1, and 2132-5 clusters together and shows the highest identity of 98.29–98.6% with the isolate Mallard/Korea/WB/KU628/2009 (JQ966084.1) collected from a wild waterfowl in Korea [32]. Isolate 226 shows the highest identity of 98.48% with the isolate Duck/China/JX/75C2/2016 (MH289831.1) collected in China [33]. Isolate 201 shows the highest identity of 98.48% with the isolate Ruddy Shelduck/Ukraine/AN/371502/2011 (KF851270.1) collected in Ukraine [34]. Isolates 1689-3-1, 1310-4-4, and 231 clusters together and shows the highest identity of 98.29–98.6% with the isolate Chicken/China/GX21/2013 (KR869090.1) collected in China [33]. Based on genetic analyses, these 10 isolates of APMV viruses obtained from various regions of Korea in this study showed high homology to the APMV-1 isolates of sub-genotype I.2 (former sub-genotype Ib) which was dominantly isolated from Russia, Japan, China, and Korea. The deduced amino acid motif at the fusion protein cleavage site of the 10 isolates of sub-genotype I.2 (former sub-genotype Ib) was ^112^GKQGRL^117^ for 7 isolates and ^112^EKQGRL^117^ for 3 isolates; these characteristics were all typical of avirulent APMV viruses.

Furthermore, three remaining isolates of APMV-1 from 2021 (209-2, 209-5, and 824-5) clustered together and showed the highest homology (more than 97%) with Mallard/USA/MD/02/217/2002 isolate (EF564824) of the sub-genotype 1.2 within class I of APMV-1 (Figure S4) [35]. The genetic identity of these viruses with the rest of the virus sub-genotype was >96%. The fusion protein cleavage site of these isolates was different at motif ^112^ERQER^117^, which is characteristic of typical avirulent APMV-1 strains.

### 3.3. Genetic Characterization of AMPV-13

The isolate of 29-9 from greater white-fronted Goose was clustered together with strains of APMV-13 mainly found in Anser albifrons from Asia and Europe. Phylogenetic analysis of the whole F gene (1638 bp) of 29-9 revealed a very close genetic relationship among these APMV-13 strains with very few genetic variations. APMV-13 29-9 isolated in this study showed a closer relationship with the strain V93-1/2015 (MN150295) recently isolated from China (97.7%) than with the APMV-13 strains obtained from Kazakhstan (96.8%), Japan (95.9%), and Ukraine (95.9%) [6,7,8,9,36,37]. The deduced amino acid sequence of the putative cleavage site of the F gene in this strain was ^112^VRENRL^117^ (Table 1), this resembled the motif, a pair of single basic residues, of the lentogenic APMV-1.

## 4. Discussion

In the present study, a total of 14 APMV strains were isolated from wild waterfowl. Of these strains, 13 strains of APMV-1 were isolated from different sites in Korea during the study period. And this result showed that APMV-1 was relatively frequently distributed in wild waterfowl species migrating to Korea from northern regions. Conversely, one strain of APMV-13, which was isolated in 2017 from wild waterfowl, was considerably rare, even at a global scale [3,7,8,9,10,36].

The phylogenetic analysis of APMV-1 isolates showed that F gene sequences clustered with those of class I and II viruses, 10 strains (71.4%) clustered within class II, and 3 (21.4%) clustered together within class I. This result corroborates previous studies reporting the predominance of class II of APMV-1 in wild waterfowls [33]. According to a recently suggested nomenclature of APMV-1 genotypes, class II is divided into 21 distinct genotypes; using this classification criterion for the phylogenetic analysis based on complete F gene sequences, the isolates from this study were determined to belong to the sub-genotype I.2 (formerly known as Ib), within this sub-genotype, most closely with APMV-1 strains from wild waterfowl in the world including Korea, and to viral sequences from birds sampled at live bird markets in Korea and China [32,38]. These results suggest that APMV-1 exchange occurs between wild waterfowls and domestic poultry species. In addition, open breeding and poor feeding conditions in Asian countries that are sites of wild waterfowl migration may contribute to the exchange of APMV-1 between wild waterfowl species and poultry [39]. Moreover, our result also confirmed the transmission possibility between wild waterfowl and poultry; the four strains (Mandarin duck/Korea/201/2016, Mallard/Korea/1310-4-4/2020, Eurasian teal/Korea/1689-3-1/2020, and Mandarin duck/Korea/231/2016) of APMV-1 found in this study showed high homology with the virus (Chicken/China/GX21/2013) isolated from chicken in China, and one strain (Mandarin duck/Korea/226/2016) had high homology with the virus strain (Duck/China/JX/75C2/2016) isolated from a duck in China. Thus, to prevent the transmission of APMV-1 among different avian species, poultry management strategies should be implemented to prevent direct and indirect contact of different poultry species with wild waterfowl. Water-borne transmission of AIV is considered an important transmission mechanism of virus dissemination [20]. Most APMV-1 strains from wild waterfowl in this study, including wild duck and goose, emphasized the extensive dissemination ability of these bird species during their migration along their flyway. Furthermore, the water supply for poultry should be clean and should not come from surface water wherein the water source could be contaminated by migratory birds.

The results of the phylogenetic analysis of selected APMV-1 showed that some Korean APMV-1 strains are highly identical to viruses from other geographical regions in the world even beyond nearby countries or the same migration flyway. This may be explained in terms of the migration of wild waterfowl into the Korean peninsula via different countries [17]. The strain of Mandarin duck/Korea/201/2016, which was obtained from Mandarin duck in this study, belongs to class II of APMV-1 and has a high degree of similarity to the virus from Ukraine (Europe), Russia (Europe/Asia), and Nigeria (Africa), which are located on different migration flyways of Black Sea/Mediterranean, Central Asian, and East Atlantic, respectively. Our results confirmed the evidence for intercontinental virus spread, particularly of APMV-1 class II genotype I sub-genotypes I.2. Furthermore, the position of APMV-1 class I sequences derived from migratory birds sampled in Korea within clades of America-origin fusion gene sequences supports the notion of intercontinental movement of birds between Asia and North America; furthermore, the finding also suggests that there is an ongoing intercontinental transmission of APMV-1 virus of North American origin into Korea, and this notion is supported by the strains of APMV-1 class I isolated in 2021 [40,41]. Our results are in agreement with previous studies that showed that wild migratory wild waterfowl were natural reservoirs of APMV-1 virus, serve as intercontinental carriers of APMV-1 virus, could be infected with different genotypes of APMV-1, and can disseminate APMV-1 to new areas [42]. To our knowledge, this is the first report of class I of APMV-1 isolated from wild waterfowl in Korea. Although class I and class II APMV-1 in this study were all lentogenic, concerns still remain regarding possible genetic changes that may happen to APMV-1 with low virulence upon replication and circulation in chickens [3]. A virulent APMV-1 of class I isolated from disease outbreaks in poultry was reported to be antigenically and genetically closely related to avirulent APMV-1 strains isolated from waterfowl [43]. In addition, genomic analysis of class II APMV-1 showed that the lentogenic virus strains can become virulent over time and that a change in the cleavage site of the fusion protein of the native virus resulted in increased virulence [44]. In addition, experimental studies have highlighted this concern; the virulence of APMV-1 virus with low initial virulence increased after several passages among poultry [45]. The APMV-1 viruses could replicate well in waterfowl and had the advantage of allowing the host to live longer, thus increasing the chances for viral replication and virus dissemination [46]. Since APMV-1 viruses with low virulence can infect a broader range of wild waterfowl species, the selection pressure on the F protein in different wild waterfowl species and different environmental stresses along the long migratory flyway may cause multiple adaptive mutations in the cleavage site. Although the adaptation mutations in the F gene of the APMV-1 virus were not fully understood, the potential risk of wild waterfowl carrying low virulence strains could not be ignored considering that only a few nucleotide changes in the F gene are sufficient to change the virulence from low to high, and countless doses of live-attenuated vaccine virus may circulate in the environment, which may exert a positive selective pressure to promote the mutation in the F gene cleavage site [47].

In this study, for the first time, we obtained a novel APMV-13 virus (greater white-fronted goose/Korea/29-9/2017) from a migratory waterfowl in Jeonbuk Province in Korea in 2017. The phylogenetic analysis revealed that the strain of APMV-13 had high homology to the viruses isolated from China, and the F gene of this virus showed some genetic diversity when compared with the virus from Kazakhstan and Ukraine. Indeed, results of previous studies suggested that Korea and China are located along this East Asian/Australasian flyway; they share the gene pool of AIV and APMV-1 and are vulnerable to the direct exchange of AIV virus via wild waterfowl [48]. Previous studies and our results suggest that a potential direct transmission of the APMV-13 virus occurred between Korea and China via wild waterfowl migration [32]. In comparison, APMV-1 has more success in terms of transmission as it is a widespread species all over the world, whereas APMV-13 is a rare species that is not involved in active circulation. Low evolutionary changes in all these APMV-13 strains potentially indicate its low circulation among wild waterfowls; these viruses are naturally endemic in wild waterfowls and do not suffer the selective pressures of vaccination or human intervention. The amino acid sequence motif 112VRENRL117 at the F protein cleavage site resembles the lentogenic APMV as reported in previous studies [7,10]. However, lentogenic APMV-1 from wild waterfowl becomes velogenic after several passages in chickens and may result in 100% mortality in the infected chickens [42]. Thus, is it important to further determine the pathogenicity and virulence of APMV-13 in poultry. APMV-13 was isolated from wild geese in this study, which corroborates previous results in other countries [7,8,9]. The dispersal and transmission of APMV-13 to other wild waterfowl species needs to be confirmed, particularly for the domestically bred avian species. As there was scale surveillance for APMV-13 in wild waterfowl and poultry, to assess the prevalence, distribution, and implication of APMV-13 to poultry health and ecology, active monitoring of APMV-13 in wild waterfowl and poultry should be implemented.

## 5. Conclusions

In conclusion, our results confirm the widespread circulation of APMV in wild waterfowl populations and that wild waterfowl play an important role in the epidemiology of APMV. The results of phylogenetic analysis based on the complete F gene sequences indicated the existence of a high APMV genetic diversity (at least APMV viruses and two different classes of APMV-1) among wild waterfowl species pathing through Korea in recent years. Our current study also demonstrates the first evidence of the existence of class I of APMV-1 and APMV-13 in wild waterfowl sampled in Korea. Our findings are helpful for understanding the genetic characteristics, epidemiology, and evolution of APMV along the East Asian–Australian migration route. The Korean isolates obtained in this study were closely related to Asian, European, and North American APMV isolates, which indicates a possible direct intercontinental transmission of the virus between these regions. Therefore, extensive surveillance should be implemented for APMV monitoring among wild waterfowl to prevent the introduction of new APMV viruses from other geographic regions.

## Figures and Tables

**Table 1:** APMVs isolate information obtained in the study.

No.	Isolate	Abbreviation	Year	Location	Species	Virus	Class	Sub-Genotype (Current/ Former)	Fusion Gene Cleavage Site (112–117)	Accession Number
1	Mandarin duck/Korea/201/2016	201	2016	Chungnam	Mandarin duck (*Aix galericulata*)	APMV-1	II	I.2/Ib	GKQGR↓L	MW464627
2	Mandarin duck/Korea/226/2016	226	2016	Chungnam	Mandarin duck (*Aix galericulata*)	APMV-1	II	I.2/Ib	GKQGR↓L	MW464628
3	Mandarin duck/Korea/231/2016	231	2016	Chungnam	Mandarin duck (*Aix galericulata*)	APMV-1	II	I.2/Ib	GKQGR↓L	MW464629
4	Mallard/Korea/92-1/2017	92-1	2017	Jeonbuk	Mallard (*Anas platyrhynchos*)	APMV-1	II	I.2/Ib	GKQGR↓L	MW464630
5	Indian spot-billed duck/Korea/92-8/2017	92-8	2017	Jeonbuk	Indian spot-billed duck (*Anas poecilorhyncha*)	APMV-1	II	I.2/Ib	GKQGR↓L	MW464631
6	Mallard/Korea/1310-4-4/2020	1310-4-4	2020	Chungnam	Mallard (*Anas platyrhynchos*)	APMV-1	II	I.2/Ib	GKQGR↓L	MW464632
7	Eurasian teal/Korea/1689-3-1/2020	1689-3-1	2020	Chungnam	Eurasian teal (*Anas crecca*)	APMV-1	II	I.2/Ib	GKQGR↓L	MW464633
8	Mandarin duck/Korea/2128-2/2020	2128-2	2020	Jeonbuk	Mandarin duck (*Aix galericulata*)	APMV-1	II	I.2/Ib	EKQGR↓L	MW464634
9	Mandarin duck/Korea/2129-1/2020	2129-1	2020	Jeonbuk	Mandarin duck (*Aix galericulata*)	APMV-1	II	I.2/Ib	EKQGR↓L	MW464635
10	Mandarin duck/Korea/2132-5/2020	2132-5	2020	Jeonbuk	Mandarin duck (*Aix galericulata*)	APMV-1	II	I.2/Ib	EKQGR↓L	MW464636
11	Taiga bean-goose/Korea/209-2/2021	209-2	2021	Jeonbuk	Taiga bean-goose (*Anser fabalis*)	APMV-1	I	1.2/1c	ERQER↓L	MZ579651
12	Taiga bean-goose/Korea/209-5/2021	209-5	2021	Jeonbuk	Taiga bean-goose (*Anser fabalis*)	APMV-1	I	1.2/1c	ERQER↓L	MZ579652
13	Greater white-fronted goose/Korea/824-5/2021	824-5	2021	Chungnam	Greater white-fronted goose (*Anser albifrons*)	APMV-1	I	1.2/1c	ERQER↓L	MZ579653
14	Greater white-fronted goose/Korea/29-9/2017	29-9	2017	Jeonbuk	Greater white-fronted goose (*Anser albifrons*)	APMV-13	-	-	VRENR↓L	MW492545

## Data Availability

All the results of this study are presented within the manuscript and its Supplementary files.

## Supplementary Materials

The following supporting information can be downloaded at: https://www.mdpi.com/article/10.3390/ani14050780/s1. Table S1: prevalence of APMV in waterfowls from Korea during 2015–2021. Table S2: seasonal prevalence of APMV in wild waterfowls from Korea during 2015–2021. Table S3: APMV isolates for phylogenetic analysis used in this study. Figure S1: locations of the areas where wild migrating birds were sampled. Figure S2: phylogenetic analyses based on the full-length nucleotide sequence of the fusion gene of isolates representing APMV 1–22 isolates. Figure S3: phylogenetic analyses based on the full-length nucleotide sequence of the fusion gene of isolates representing APMV-1 class II isolates. Figure S4: phylogenetic analyses based on the full-length nucleotide sequence of the fusion gene of isolates representing APMV-1 class I isolates.
