# Supplementary material for: Genetic Characterization of Avian Paramyxovirus Isolated from Wild Waterfowl in Korea between 2015 and 2021
Source: Animals (Basel). 2024 Mar 1;14(5):780. doi: 10.3390/ani14050780 (PMC10930869; doi:10.3390/ani14050780)
Supplement: Supplementary file 1 [file animals-14-00780-s001.zip › Fig S1.pdf]

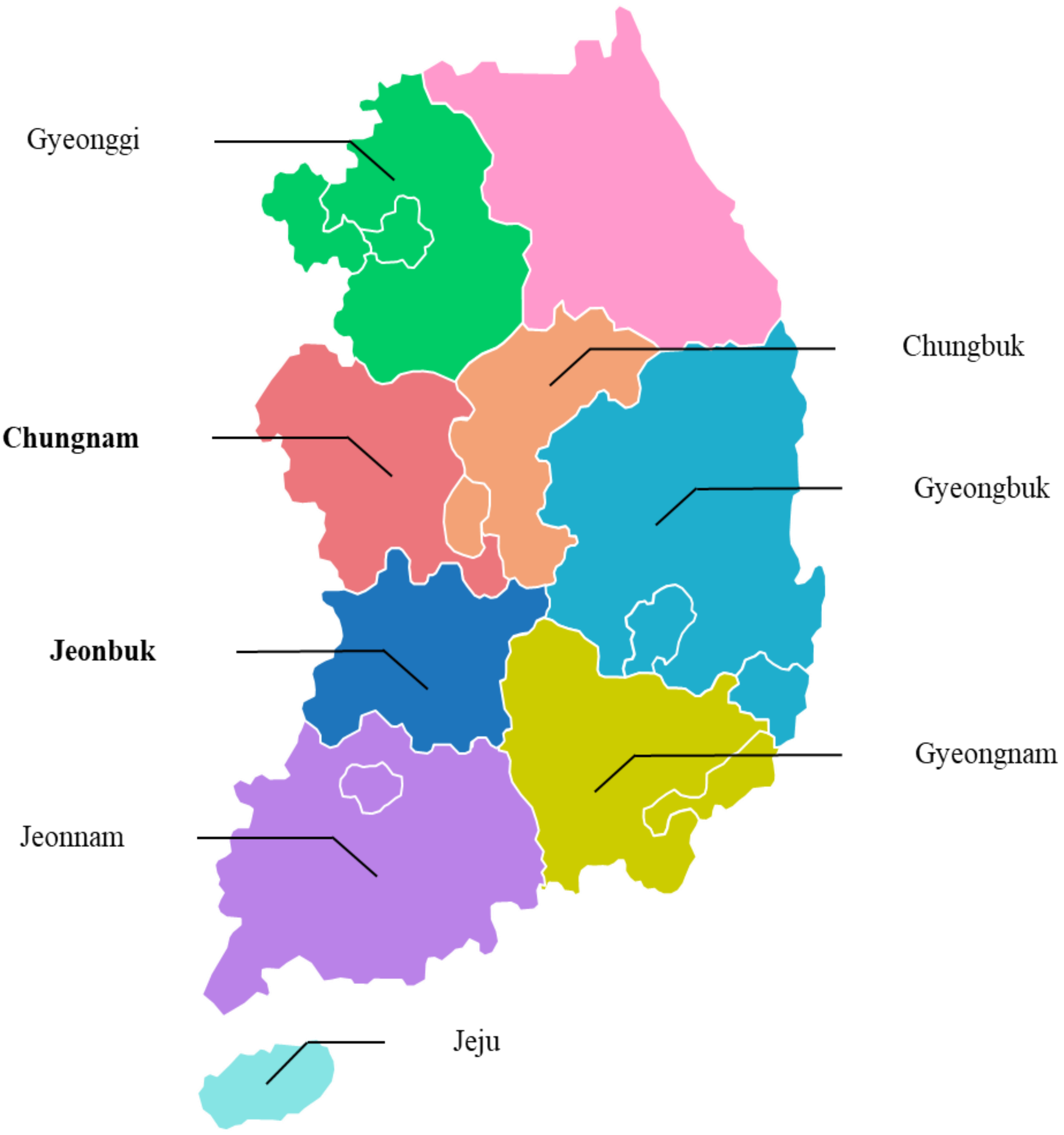

Figure S1 | Locations of the areas where wild migrating birds were sampled. Regions where positive samples were detected are designated in bold.
