# Supplementary material for: Genetic Characterization of Avian Paramyxovirus Isolated from Wild Waterfowl in Korea between 2015 and 2021
Source: Animals (Basel). 2024 Mar 1;14(5):780. doi: 10.3390/ani14050780 (PMC10930869; doi:10.3390/ani14050780)
Supplement: Supplementary file 1 [file animals-14-00780-s001.zip › Fig S2.pdf]

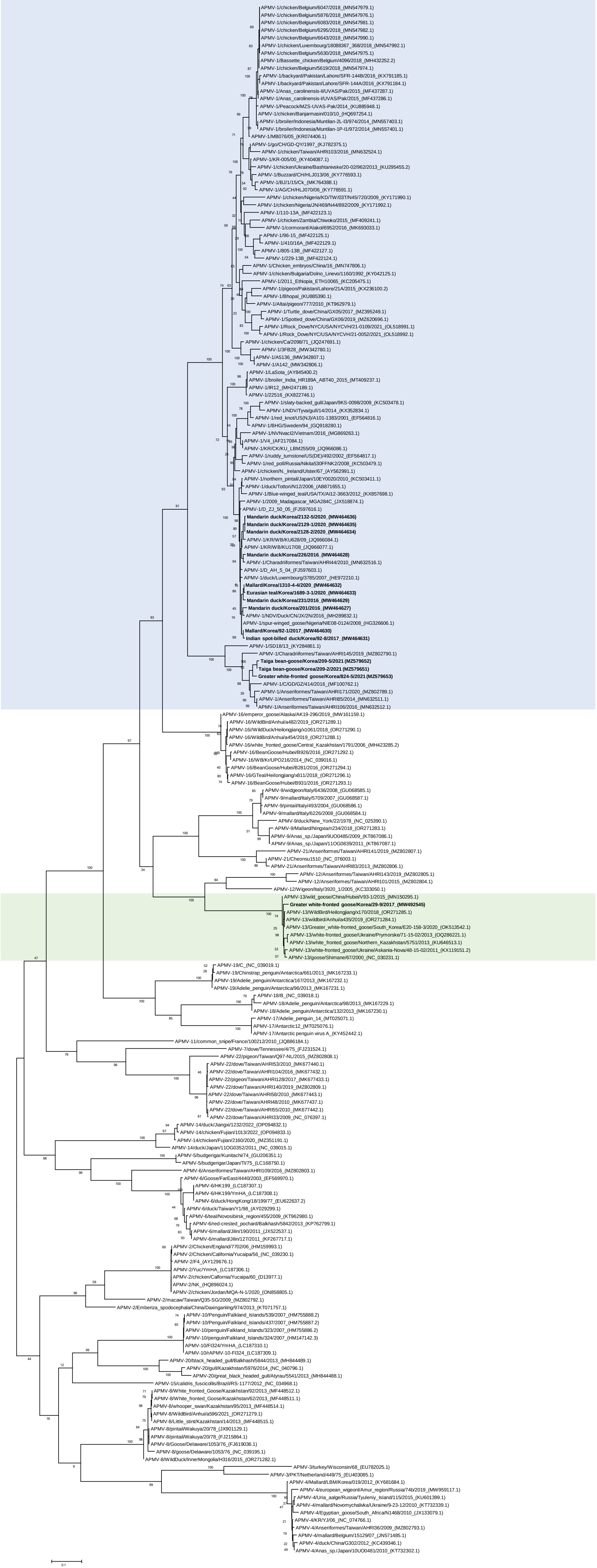

APMV-1/chicken/Belgium/6047/2018_(MN547979.1)
APMV-1/chicken/Belgium/5876/2018_(MN547976.1)
APMV-1/chicken/Belgium/6083/2018_(MN547981.1)
APMV-1/chicken/Belgium/6295/2018_(MN547982.1)
APMV-1/chicken/Belgium/6643/2018_(MN547990.1)
APMV-1/chicken/Luxembourg/18088367_368/2018_(MN547992.1)
APMV-1/chicken/Belgium/5630/2018_(MN547975.1)
APMV-1/Bassette_chicken/Belgium/4096/2018_(MH432252.2)
APMV-1/chicken/Belgium/5619/2018_(MN547974.1)
APMV-1/backyard/Pakistan/Lahore/SFR-144B/2016_(KX791185.1)
APMV-1/backyard/Pakistan/Lahore/SFR-144A/2016_(KX791184.1)
APMV-1/Anas_carolinensis-II/UVAS/Pak/2015_(MF437287.1)
APMV-1/Anas_carolinensis-I/UVAS/Pak/2015_(MF437286.1)
APMV-1/Peacock/MZS-UVAS-Pak/2014_(KU885948.1)
APMV-1/chicken/Banjarmasin/010/10_(HQ697254.1)
APMV-1/broiler/Indonesia/Muntilan-2L-I3/974/2014_(MN557403.1)
APMV-1/broiler/Indonesia/Muntilan-1P-I1/972/2014_(MN557401.1)
APMV-1/MB076/05_(KR074406.1)
APMV-1/go/CH/GD-QY/1997_(KJ782375.1)
APMV-1/chicken/Taiwan/AHRI103/2016_(MN632524.1)
APMV-1/KR-005/00_(KY404087.1)
APMV-1/chicken/Ukraine/Bashtanivske/20-02/962/2013_(KU295455.2)
APMV-1/Buzzard/CH/HLJ013/06_(KY776593.1)
APMV-1/BJ/1/15/Ck_(MK764388.1)
APMV-1/AG/CH/HLJ070/06_(KY776591.1)
APMV-1/chicken/Nigeria/KD/TW/03T/N45/720/2009_(KY171990.1)
APMV-1/chicken/Nigeria/JN/469/N44/892/2009_(KY171992.1)
APMV-1/110-13A_(MF422123.1)
APMV-1/chicken/Zambia/Chiwoko/2015_(MF409241.1)
APMV-1/cormorant/Alakol/6952/2016_(MK693033.1)
APMV-1/96-15_(MF422125.1)
APMV-1/410/16A_(MF422129.1)
APMV-1/805-13B_(MF422127.1)
APMV-1/229-13B_(MF422124.1)
APMV-1/Chicken_embryos/China/16_(MN747806.1)
APMV-1/chicken/Bulgaria/Dolno_Linevo/1160/1992_(KY042125.1)
APMV-1/2011_Ethiopia_ETH10065_(KC205475.1)
APMV-1/pigeon/Pakistan/Lahore/21A/2015_(KX236100.2)
APMV-1/Bhopal_(KU885390.1)
APMV-1/Altai/pigeon/777/2010_(KT962979.1)
APMV-1/Turtle_dove/China/GX05/2017_(MZ395249.1)
APMV-1/Spotted_dove/China/GX06/2019_(MZ620696.1)
APMV-1/Rock_Dove/NYC/USA/NYCVH/21-0109/2021_(OL518991.1)
APMV-1/Rock_Dove/NYC/USA/NYCVH/21-0052/2021_(OL518992.1)
APMV-1/chicken/Ca/2098/71_(JQ247691.1)
APMV-1/3FB28_(MW342780.1)
APMV-1/A5136_(MW342807.1)
APMV-1/A142_(MW342806.1)
APMV-1/LaSota_(AY845400.2)
APMV-1/broiler_India_HR189A_ABT40_2015_(MT409237.1)
APMV-1/IR12_(MH247189.1)
APMV-1/22516_(KX822746.1)
APMV-1/slaty-backed_gull/Japan/9KS-0098/2009_(KC503478.1)
APMV-1/NDV/Tyva/gull/14/2014_(KX352834.1)
APMV-1/red_knot/US(NJ)/A101-1383/2001_(EF564816.1)
APMV-1/BHG/Sweden/94_(GQ918280.1)
APMV-1/NVNvacl2/Vietnam/2016_(MG869263.1)
APMV-1/V4_(AF217084.1)
APMV-1/KR/CK/KU_LBM255/09_(JQ966086.1)
APMV-1/ruddy_turnstone/US(DE)/492/2002_(EF564817.1)
APMV-1/red_poll/Russia/Nikita530FFNK2/2008_(KC503479.1)
APMV-1/chicken/N._Ireland/Ulster/67_(AY562991.1)
APMV-1/northern_pintail/Japan/10EY0020/2010_(KC503411.1)
APMV-1/duck/Tottori/N12/2006_(AB871655.1)
APMV-1/Blue-winged_teal/USA/TX/AI12-3663/2012_(KX857698.1)
APMV-1/2009_Madagascar_MGA284C_(JX518874.1)
APMV-1/D_ZJ_50_05_(FJ597616.1)
Mandarin duck/Korea/2132-5/2020_(MW464636)
Mandarin duck/Korea/2129-1/2020_(MW464635)
Mandarin duck/Korea/2128-2/2020_(MW464634)
APMV-1/KR/WB/KU628/09_(JQ966084.1)
APMV-1/KR/WB/KU17/08_(JQ966077.1)
Mandarin duck/Korea/226/2016_(MW464628)
APMV-1/Charadriiformes/Taiwan/AHRI44/2010_(MN632516.1)
APMV-1/D_AH_5_04_(FJ597603.1)
APMV-1/duck/Luxembourg/3785/2007_(HE972210.1)
Mallard/Korea/1310-4-4/2020_(MW464632)
Eurasian teal/Korea/1689-3-1/2020_(MW464633)
Mandarin duck/Korea/231/2016_(MW464629)
Mandarin duck/Korea/201/2016_(MW464627)
APMV-1/NDV/Duck/CN/JX/2N/2016_(MH289832.1)
APMV-1/spur-winged_goose/Nigeria/NIE08-0124/2008_(HG326606.1)
Mallard/Korea/92-1/2017_(MW464630)
Indian spot-billed duck/Korea/92-8/2017_(MW464631)
APMV-1/SD18/13_(KY284861.1)
APMV-1/Charadriiformes/Taiwan/AHRI145/2019_(MZ802790.1)
Taiga bean-goose/Korea/209-5/2021 (MZ579652)
Taiga bean-goose/Korea/209-2/2021 (MZ579651)
Greater white-fronted goose/Korea/824-5/2021 (MZ579653)
APMV-1/C/GD/GZ/414/2016_(MF100762.1)
APMV-1/Anseriformes/Taiwan/AHRI171/2020_(MZ802789.1)
APMV-1/Anseriformes/Taiwan/AHRI85/2014_(MN632511.1)
APMV-1/Anseriformes/Taiwan/AHRI106/2016_(MN632512.1)
APMV-16/emperor_goose/Alaska/AK19-296/2019_(MW161159.1)
APMV-16/WildBird/Anhui/a482/2019_(OR271289.1)
APMV-16//WildDuck/Heilongjiang/x1061/2018_(OR271290.1)
APMV-16/WildBird/Anhui/a454/2019_(OR271288.1)
APMV-16/white_fronted_goose/Central_Kazakhstan/1791/2006_(MH423285.2)
APMV-16/BeanGoose/Hubei/B926/2016_(OR271292.1)
APMV-16/WB/Kr/UPO216/2014_(NC_039016.1)
APMV-16/BeanGoose/Hubei/B281/2016_(OR271294.1)
APMV-16/GTeal/Heilongjiang/x811/2018_(OR271296.1)
APMV-16/BeanGoose/Hubei/B931/2016_(OR271293.1)
APMV-9/widgeon/Italy/6436/2008_(GU068585.1)
APMV-9/mallard/Italy/5709/2007_(GU068587.1)
APMV-9/pintail/Italy/493/2004_(GU068586.1)
APMV-9/mallard/Italy/6226/2008_(GU068584.1)
APMV-9/duck/New_York/22/1978_(NC_025390.1)
APMV-9/Mallard/Ningxia/n234/2018_(OR271283.1)
APMV-9/Anas_sp./Japan/9UO0485/2009_(KT867086.1)
APMV-9/Anas_sp./Japan/11OG0839/2011_(KT867087.1)
APMV-21/Anseriformes/Taiwan/AHRI141/2019_(MZ802807.1)
APMV-21/Cheonsu1510_(NC_076003.1)
APMV-21/Anseriformes/Taiwan/AHRI83/2013_(MZ802806.1)
APMV-12/Anseriformes/Taiwan/AHRI143/2019_(MZ802805.1)
APMV-12/Anseriformes/Taiwan/AHRI101/2015_(MZ802804.1)
APMV-12/Wigeon/Italy/3920_1/2005_(KC333050.1)
APMV-13/wild_goose/China/Hubei/V93-1/2015_(MN150295.1)
Greater white-fronted goose/Korea/29-9/2017_(MW492545)
APMV-13/WildBird/Heilongjiang/x170/2018_(OR271285.1)
APMV-13/wildbird/Anhui/a435/2019_(OR271284.1)
APMV-13/Greater_white-fronted_goose/South_Korea/E20-158-3/2020_(OK513542.1)
APMV-13/white-fronted_goose/Ukraine/Prymorske/71-15-02/2013_(OQ286221.1)
APMV-13/white_fronted_goose/Northern_Kazakhstan/5751/2013_(KU646513.1)
APMV-13/white-fronted_goose/Ukraine/Askania-Nova/48-15-02/2011_(KX119151.2)
APMV-13/goose/Shimane/67/2000_(NC_030231.1)
APMV-19/C_(NC_039019.1)
APMV-19/Chinstrap_penguin/Antarctica/661/2013_(MK167233.1)
APMV-19/Adelie_penguin/Antarctica/167/2013_(MK167232.1)
APMV-19/Adelie_penguin/Antarctica/96/2013_(MK167231.1)
APMV-18/B_(NC_039018.1)
APMV-18/Adelie_penguin/Antarctica/98/2013_(MK167229.1)
APMV-18/Adelie_penguin/Antarctica/132/2013_(MK167230.1)
APMV-17/Adelie_penguin_14_(MT025071.1)
APMV-17/Antarctic12_(MT025076.1)
APMV-17/Antarctic penguin virus A_(KY452442.1)
APMV-11/common_snipe/France/100212/2010_(JQ886184.1)
APMV-7/dove/Tennessee/4/75_(FJ231524.1)
APMV-22/pigeon/Taiwan/Q97-NL/2015_(MZ802808.1)
APMV-22/dove/Taiwan/AHRI53/2010_(MK677440.1)
APMV-22/dove/Taiwan/AHRI104/2016_(MK677432.1)
APMV-22/pigeon/Taiwan/AHRI128/2017_(MK677433.1)
APMV-22/dove/Taiwan/AHRI140/2019_(MZ802809.1)
APMV-22/dove/Taiwan/AHRI58/2010_(MK677443.1)
APMV-22/dove/Taiwan/AHRI48/2010_(MK677437.1)
APMV-22/dove/Taiwan/AHRI55/2010_(MK677442.1)
APMV-22/dove/Taiwan/AHRI33/2009_(NC_076397.1)
APMV-14/duck/Jiangxi/1232/2022_(OP094832.1)
APMV-14/chicken/Fujian/1013/2022_(OP094833.1)
APMV-14/chicken/Fujian/2160/2020_(MZ351191.1)
APMV-14/duck/Japan/11OG0352/2011_(NC_039015.1)
APMV-5/budgerigar/Kunitachi/74_(GU206351.1)
APMV-5/budgerigar/Japan/TI/75_(LC168750.1)
APMV-6/Anseriformes/Taiwan/AHRI109/2016_(MZ802803.1)
APMV-6/Goose/FarEast/4440/2003_(EF569970.1)
APMV-6/HK199_(LC187307.1)
APMV-6/HK199/YmHA_(LC187308.1)
APMV-6/duck/HongKong/18/199/77_(EU622637.2)
APMV-6/duck/Taiwan/Y1/98_(AY029299.1)
APMV-6/teal/Novosibirsk_region/455/2009_(KT962980.1)
APMV-6/red-crested_pochard/Balkhash/5842/2013_(KP762799.1)
APMV-6/mallard/Jilin/190/2011_(JX522537.1)
APMV-6/mallard/Jilin/127/2011_(KF267717.1)
APMV-2/Chicken/England/7702/06_(HM159993.1)
APMV-2/Chicken/California/Yucaipa/56_(NC_039230.1)
APMV-2/F4_(AY129676.1)
APMV-2/Yuc/YmHA_(LC187306.1)
APMV-2/chicken/Calfornia/Yucaipa/60_(D13977.1)
APMV-2/NK_(HQ896024.1)
APMV-2/chicken/Jordan/MQA-N-1/2020_(ON858805.1)
APMV-2/macaw/Taiwan/Q35-SG/2009_(MZ802792.1)
APMV-2/Emberiza_spodocephala/China/Daxinganling/974/2013_(KT071757.1)
APMV-10/Penguin/Falkland_Islands/539/2007_(HM755888.2)
APMV-10/Penguin/Falkland_Islands/437/2007_(HM755887.2)
APMV-10/penguin/Falkland_Islands/323/2007_(HM755886.2)
APMV-10/penguin/Falkland_Islands/324/2007_(HM147142.3)
APMV-10/FI324/YmHA_(LC187310.1)
APMV-10/rAPMV-10-FI324_(LC187309.1)
APMV-20/black_headed_gull/Balkhash/5844/2013_(MH844489.1)
APMV-20/gull/Kazakhstan/5976/2014_(NC_040796.1)
APMV-20/great_black_headed_gull/Atyrau/5541/2013_(MH844488.1)
APMV-15/calidris_fuscicollis/Brazil/RS-1177/2012_(NC_034968.1)
APMV-8/White_fronted_Goose/Kazakhstan/92/2013_(MF448512.1)
APMV-8/White_fronted_Goose/Kazakhstan/62/2013_(MF448511.1)
APMV-8/whooper_swan/Kazakhstan/95/2013_(MF448514.1)
APMV-8/WildBird/Anhui/a596/2021_(OR271279.1)
APMV-8/Little_stint/Kazakhstan/14/2013_(MF448515.1)
APMV-8/pintail/Wakuya/20/78_(JX901129.1)
APMV-8/pintail/Wakuya/20/78_(FJ215864.1)
APMV-8/Goose/Delaware/1053/76_(FJ619036.1)
APMV-8/goose/Delaware/1053/76_(NC_039195.1)
APMV-8/WildDuck/InnerMongolia/H316/2015_(OR271282.1)
APMV-3/turkey/Wisconsin/68_(EU782025.1)
APMV-3/PKT/Netherland/449/75_(EU403085.1)
APMV-4/Mallard/LBM/Korea/019/2012_(KY681684.1)
APMV-4/european_wigeon/Amur_region/Russia/74b/2019_(MW959117.1)
APMV-4/Uria_aalge/Russia/Tyuleniy_Island/115/2015_(KU601399.1)
APMV-4/mallard/Novomychalivka/Ukraine/9-23-12/2010_(KT732339.1)
APMV-4/Egyptian_goose/South_Africa/N1468/2010_(JX133079.1)
APMV-4/KR/YJ/06_(NC_074766.1)
APMV-4/Anseriformes/Taiwan/AHRI36/2009_(MZ802793.1)
APMV-4/mallard/Belgium/15129/07_(JN571485.1)
APMV-4/duck/China/G302/2012_(KC439346.1)
APMV-4/Anas_sp./Japan/10UO0481/2010_(KT732302.1)
0.1

Figure S2 | Phylogenetic analysis was conducted based on complete nucleotide sequences of fusion genes obtained from representative isolates of Avian paramyxovirus (APMV) serotypes 1-22. Viruses described in this study are designated in bold. The new (decimal naming) and the former names (alpha-numerical) of genotype or sub-genotype in APMV-1 are provided for easier comparison. The serotypes to which the newly isolated virus belongs are color-highlighted for emphasis.
