# Supplementary material for: Genetic Characterization of Avian Paramyxovirus Isolated from Wild Waterfowl in Korea between 2015 and 2021
Source: Animals (Basel). 2024 Mar 1;14(5):780. doi: 10.3390/ani14050780 (PMC10930869; doi:10.3390/ani14050780)
Supplement: Supplementary file 1 [file animals-14-00780-s001.zip › Fig S4.pdf]

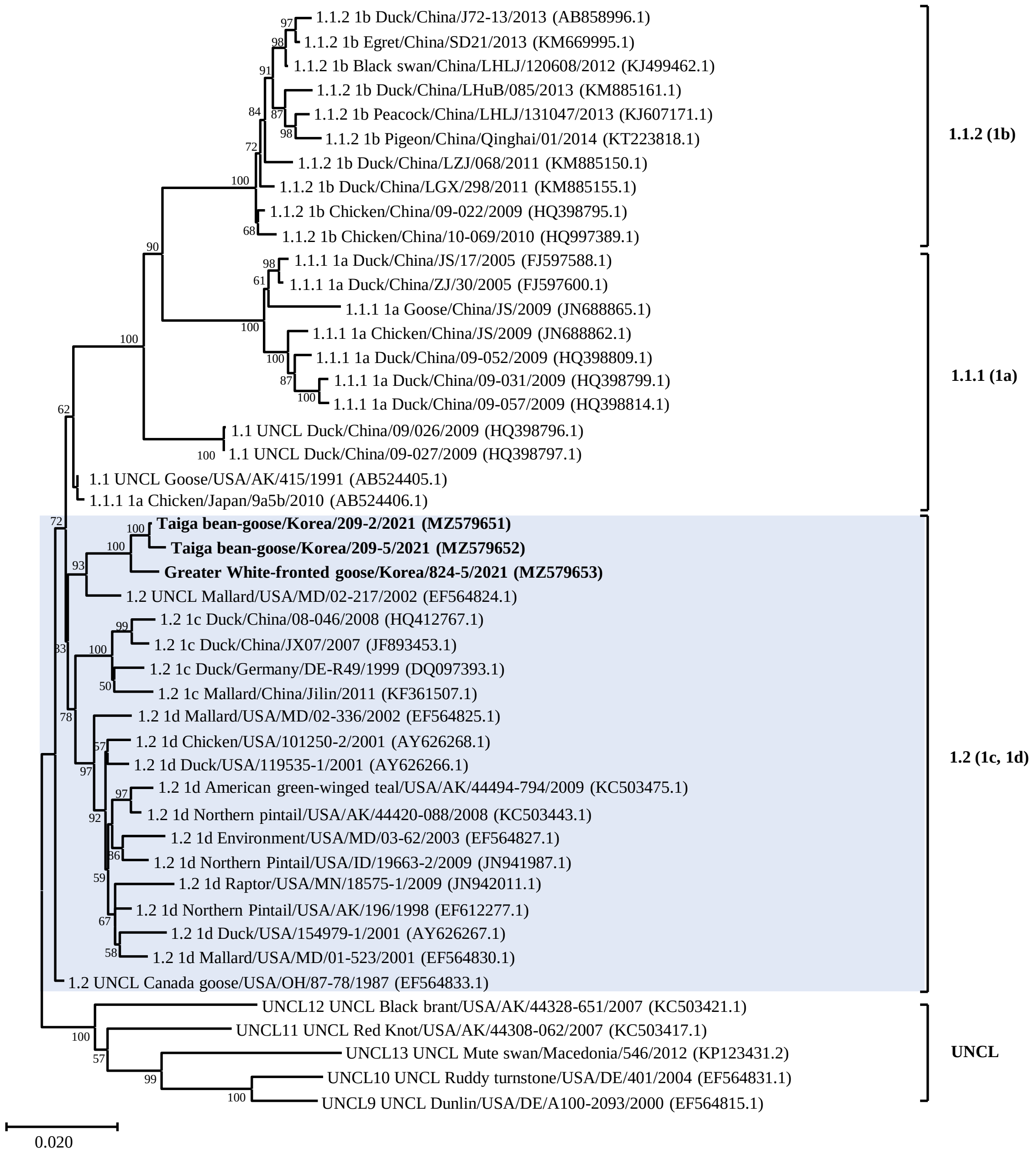

Figure S4 | Phylogenetic analyses based on the full-length nucleotide sequence of the fusion gene of isolates representing APMV-1 class I isolates. Viruses described in this study are designated in bold. The new (decimal naming) and the former names (alpha-numerical) of genotype or sub-genotype in APMV-1 are provided for easier comparison. The genotypes to which the newly isolated virus belongs are color-highlighted for emphasis.
