# Supplementary material for: Genetic Characterization of Avian Paramyxovirus Isolated from Wild Waterfowl in Korea between 2015 and 2021
Source: Animals (Basel). 2024 Mar 1;14(5):780. doi: 10.3390/ani14050780 (PMC10930869; doi:10.3390/ani14050780)
Supplement: Supplementary file 1 [file animals-14-00780-s001.zip › Supplementary Material (Table S1-S3).pdf]

Supplementary Materials

Table S1. Prevalence of APMVs in waterfowls from Korea during 2015-2021[*].

**Year**	No. of samples	No. of isolates	Positive rate (%)
**2015**	382	-	-
**2016**	127	3	2.4
**2017**	188	3	1.6
**2018**	256	-	-
**2019**	207	-	-
**2020**	331	5	1.5
**2021**	45	3	6.7
**Total**	1,536	14	0.9

[*], the 14 isolates were HA and RT-PCR positive for APMV; -, no samples were included.

Table S2. Seasonal prevalence of APMVs in wild waterfowls from Korea during 2015-2021 [*].

Season	No. of samples	No. of isolates	Positive rate (%)
Spring	223	1	0.4
Summer	85	-	-
Autumn	534	10	1.9
Winter	694	3	0.4

[*], the 14 isolates were HA and RT-PCR positive for APMV; -, no samples were included.

Table S3. APMV isolates for Phylogenetic Analysis.

No.	Virus	Genus	Strains	Accession number
1	Avian paramyxovirus 1(APMV-1)	*Orthoavulavirus*	AG/CH/HLJ070/06	KY776591.1
2	Avian paramyxovirus 1(APMV-1)	*Orthoavulavirus*	go/CH/GD-QY/1997	KJ782375.1
3	Avian paramyxovirus 1(APMV-1)	*Orthoavulavirus*	MB076/05	KR074406.1
4	Avian paramyxovirus 1(APMV-1)	*Orthoavulavirus*	chicken/Banjarmasin/010/10	HQ697254.1
5	Avian paramyxovirus 1(APMV-1)	*Orthoavulavirus*	Peacock/MZS-UVAS-Pak/2014	KU885948.1
6	Avian paramyxovirus 1(APMV-1)	*Orthoavulavirus*	Anas carolinensis-II/UVAS/Pak/2015	MF437287.1
7	Avian paramyxovirus 1(APMV-1)	*Orthoavulavirus*	Anas carolinensis-I/UVAS/Pak/2015	MF437286.1
8	Avian paramyxovirus 1(APMV-1)	*Orthoavulavirus*	Altai/pigeon/777/2010	KT962979.1
9	Avian paramyxovirus 1(APMV-1)	*Orthoavulavirus*	AAvV-1/cormorant/Alakol/6952/2016	MK693033.1
10	Avian paramyxovirus 1(APMV-1)	*Orthoavulavirus*	96-15	MF422125.1
11	Avian paramyxovirus 1(APMV-1)	*Orthoavulavirus*	110-13A	MF422123.1
12	Avian paramyxovirus 1(APMV-1)	*Orthoavulavirus*	805-13B	MF422127.1
13	Avian paramyxovirus 1(APMV-1)	*Orthoavulavirus*	APMV-1/chicken/Ca/2098/71	JQ247691.1
14	Avian paramyxovirus 1(APMV-1)	*Orthoavulavirus*	410/16A	MF422129.1
15	Avian paramyxovirus 1(APMV-1)	*Orthoavulavirus*	229-13B	MF422124.1
16	Avian paramyxovirus 1(APMV-1)	*Orthoavulavirus*	pigeon/Pakistan/Lahore/21A/2015	KX236100.2
17	Avian paramyxovirus 1(APMV-1)	*Orthoavulavirus*	V4	AF217084.1
18	Avian paramyxovirus 1(APMV-1)	*Orthoavulavirus*	A5136	MW342807.1
19	Avian paramyxovirus 1(APMV-1)	*Orthoavulavirus*	A142	MW342806.1
20	Avian paramyxovirus 1(APMV-1)	*Orthoavulavirus*	3FB28	MW342780.1
21	Avian paramyxovirus 1(APMV-1)	*Orthoavulavirus*	22516	KX822746.1
22	Avian paramyxovirus 1(APMV-1)	*Orthoavulavirus*	IR12	MH247189.1
23	Avian paramyxovirus 1(APMV-1)	*Orthoavulavirus*	LaSota	AY845400.2
24	Avian paramyxovirus 1(APMV-1)	*Orthoavulavirus*	KR/CK/KU LBM255/09	JQ966086.1
25	Avian paramyxovirus 1(APMV-1)	*Orthoavulavirus*	chicken/N. Ireland/Ulster/67	AY562991.1
26	Avian paramyxovirus 1(APMV-1)	*Orthoavulavirus*	Anseriformes/Taiwan/AHRI106/2016	MN632512.1
27	Avian paramyxovirus 1(APMV-1)	*Orthoavulavirus*	Anseriformes/Taiwan/AHRI85/2014	MN632511.1
28	Avian paramyxovirus 1(APMV-1)	*Orthoavulavirus*	AOAV-1/chicken/Belgium/6643/2018	MN547990.1
29	Avian paramyxovirus 1(APMV-1)	*Orthoavulavirus*	AOAV-1/chicken/Belgium/6295/2018	MN547982.1
30	Avian paramyxovirus 1(APMV-1)	*Orthoavulavirus*	AOAV-1/chicken/Belgium/5876/2018	MN547976.1
31	Avian paramyxovirus 1(APMV-1)	*Orthoavulavirus*	AOAV-1/chicken/Belgium/5630/2018	MN547975.1
32	Avian paramyxovirus 1(APMV-1)	*Orthoavulavirus*	AOAV-1/chicken/Luxembourg/18088367_368/2018	MN547992.1
33	Avian paramyxovirus 1(APMV-1)	*Orthoavulavirus*	AOAV-1/chicken/Belgium/6083/2018	MN547981.1
34	Avian paramyxovirus 1(APMV-1)	*Orthoavulavirus*	AOAV-1/chicken/Belgium/6047/2018	MN547979.1
35	Avian paramyxovirus 1(APMV-1)	*Orthoavulavirus*	AOAV-1/chicken/Belgium/5619/2018	MN547974.1
36	Avian paramyxovirus 1(APMV-1)	*Orthoavulavirus*	APMV-1/Anseriformes/Taiwan/AHRI171/2020	MZ802789.1
37	Avian paramyxovirus 1(APMV-1)	*Orthoavulavirus*	APMV1/Bassette chicken/Belgium/4096/2018	MH432252.2
38	Avian paramyxovirus 1(APMV-1)	*Orthoavulavirus*	APMV-1/Charadriiformes/Taiwan/AHRI145/2019	MZ802790.1

39	Avian paramyxovirus 1(APMV-1)	*Orthoavulavirus*	APMV-1/Rock Dove/NYC/USA/NYCVH/21-0052/2021	OL518992.1
40	Avian paramyxovirus 1(APMV-1)	*Orthoavulavirus*	APMV-1/Rock Dove/NYC/USA/NYCVH/21-0109/2021	OL518991.1
41	Avian paramyxovirus 1(APMV-1)	*Orthoavulavirus*	backyard/Pakistan/Lahore/SFR-144A/2016	KX791184.1
42	Avian paramyxovirus 1(APMV-1)	*Orthoavulavirus*	backyard/Pakistan/Lahore/SFR-144B/2016	KX791185.1
43	Avian paramyxovirus 1(APMV-1)	*Orthoavulavirus*	Bhopal	KU885390.1
44	Avian paramyxovirus 1(APMV-1)	*Orthoavulavirus*	BJ/1/15/Ck	MK764388.1
45	Avian paramyxovirus 1(APMV-1)	*Orthoavulavirus*	broiler/Indonesia/Muntilan-1P-I1/972/2014	MN557401.1
46	Avian paramyxovirus 1(APMV-1)	*Orthoavulavirus*	broiler/Indonesia/Muntilan-2L-I3/974/2014	MN557403.1
47	Avian paramyxovirus 1(APMV-1)	*Orthoavulavirus*	broiler_India_HR189A_ABT40_2015	MT409237.1
48	Avian paramyxovirus 1(APMV-1)	*Orthoavulavirus*	Buzzard/CH/HLJ013/06	KY776593.1
49	Avian paramyxovirus 1(APMV-1)	*Orthoavulavirus*	C/GD/GZ/414/2016	MF100762.1
50	Avian paramyxovirus 1(APMV-1)	*Orthoavulavirus*	Charadriiformes/Taiwan/AHRI44/2010	MN632516.1
51	Avian paramyxovirus 1(APMV-1)	*Orthoavulavirus*	Chicken embryos/China/16	MN747806.1
52	Avian paramyxovirus 1(APMV-1)	*Orthoavulavirus*	chicken/Bulgaria/Dolno_Linevo/1160/1992	KY042125.1
53	Avian paramyxovirus 1(APMV-1)	*Orthoavulavirus*	chicken/Nigeria/JN/469/N44/892/2009	KY171992.1
54	Avian paramyxovirus 1(APMV-1)	*Orthoavulavirus*	chicken/Nigeria/KD/TW/03T/N45/720/2009	KY171990.1
55	Avian paramyxovirus 1(APMV-1)	*Orthoavulavirus*	chicken/Taiwan/AHRI103/2016	MN632524.1
56	Avian paramyxovirus 1(APMV-1)	*Orthoavulavirus*	chicken/Ukraine/Bashtanivske/20-02/962/2013	KU295455.2
57	Avian paramyxovirus 1(APMV-1)	*Orthoavulavirus*	chicken/Zambia/Chiwoko/2015	MF409241.1
58	Avian paramyxovirus 1(APMV-1)	*Orthoavulavirus*	Turtle dove/China/GX05/2017	MZ395249.1
59	Avian paramyxovirus 1(APMV-1)	*Orthoavulavirus*	Spotted dove/China/GX06/2019	MZ620696.1
60	Avian paramyxovirus 1(APMV-1)	*Orthoavulavirus*	APMV-1/duck/Tottori/N12/2006	AB871655.1
61	Avian paramyxovirus 1(APMV-1)	*Orthoavulavirus*	AAvV1-NVNvacI2/Vietnam/2016	MG869263.1
62	Avian paramyxovirus 1(APMV-1)	*Orthoavulavirus*	red knot/US(NJ)/A101-1383/2001	EF564816.1
63	Avian paramyxovirus 1(APMV-1)	*Orthoavulavirus*	BHG/Sweden/94	GQ918280.1
64	Avian paramyxovirus 1(APMV-1)	*Orthoavulavirus*	slaty-backed gull/Japan/9KS-0098/2009	KC503478.1
65	Avian paramyxovirus 1(APMV-1)	*Orthoavulavirus*	NDV/Tyva/gull/14/2014	KX352834.1
66	Avian paramyxovirus 1(APMV-1)	*Orthoavulavirus*	ruddy turnstone/US(DE)/492/2002	EF564817.1
67	Avian paramyxovirus 1(APMV-1)	*Orthoavulavirus*	red poll/Russia/Nikita530FFNK2/2008	KC503479.1
68	Avian paramyxovirus 1(APMV-1)	*Orthoavulavirus*	D_AH_5_04	FJ597603.1
69	Avian paramyxovirus 1(APMV-1)	*Orthoavulavirus*	D_ZJ_50_05	FJ597616.1
70	Avian paramyxovirus 1(APMV-1)	*Orthoavulavirus*	APMV-1/duck/Luxembourg/3785/2007	HE972210.1
71	Avian paramyxovirus 1(APMV-1)	*Orthoavulavirus*	spur-winged goose/Nigeria/NIE08-0124/2008	HG326606.1
72	Avian paramyxovirus 1(APMV-1)	*Orthoavulavirus*	KR/WB/KU17/08	JQ966077.1
73	Avian paramyxovirus 1(APMV-1)	*Orthoavulavirus*	KR/WB/KU628/09	JQ966084.1
74	Avian paramyxovirus 1(APMV-1)	*Orthoavulavirus*	2009_Madagascar_MGA284C	JX518874.1
75	Avian paramyxovirus 1(APMV-1)	*Orthoavulavirus*	northern pintail/Japan/10EY0020/2010	KC503411.1
76	Avian paramyxovirus 1(APMV-1)	*Orthoavulavirus*	Blue-winged teal/USA/TX/AI12-3663/2012	KX857698.1
77	Avian paramyxovirus 1(APMV-1)	*Orthoavulavirus*	NDV/Duck/CN/JX/2N/2016	MH289832.1
78	Avian paramyxovirus 1(APMV-1)	*Orthoavulavirus*	KR-005/00	KY404087.1
79	Avian paramyxovirus 1(APMV-1)	*Orthoavulavirus*	2011_Ethiopia_ETH10065	KC205475.1

80	Avian paramyxovirus 1(APMV-1)	*Orthoavulavirus*	SD18/13	KY284861.1
81	Avian paramyxovirus 1(APMV-1)	*Orthoavulavirus*	Mandarin duck/Korea/2132-5/2020	MW464636
82	Avian paramyxovirus 1(APMV-1)	*Orthoavulavirus*	Mandarin duck/Korea/2129-1/2020	MW464635
83	Avian paramyxovirus 1(APMV-1)	*Orthoavulavirus*	Mandarin duck/Korea/2128-2/2020	MW464634
84	Avian paramyxovirus 1(APMV-1)	*Orthoavulavirus*	Eurasian teal/Korea/1689-3-1/2020	MW464633
85	Avian paramyxovirus 1(APMV-1)	*Orthoavulavirus*	Mallard/Korea/1310-4-4/2020	MW464632
86	Avian paramyxovirus 1(APMV-1)	*Orthoavulavirus*	Indian spot-billed duck/Korea/92-8/2017	MW464631
87	Avian paramyxovirus 1(APMV-1)	*Orthoavulavirus*	Mallard/Korea/92-1/2017	MW464630
88	Avian paramyxovirus 1(APMV-1)	*Orthoavulavirus*	Mandarin duck/Korea/231/2016	MW464629
89	Avian paramyxovirus 1(APMV-1)	*Orthoavulavirus*	Mandarin duck/Korea/226/2016	MW464628
90	Avian paramyxovirus 1(APMV-1)	*Orthoavulavirus*	Mandarin duck/Korea/201/2016	MW464627
91	Avian paramyxovirus 1(APMV-1)	*Orthoavulavirus*	Taiga bean-goose/Korea/209-2/2021	MZ579651
92	Avian paramyxovirus 1(APMV-1)	*Orthoavulavirus*	Taiga bean-goose/Korea/209-5/2021	MZ579652
93	Avian paramyxovirus 1(APMV-1)	*Orthoavulavirus*	Greater white-fronted goose/Korea/824-5/2021	MZ579653
94	Avian paramyxovirus 2(APMV-2)	*Metaavulavirus*	APMV-2/Chicken/England/7702/06	HM159993.1
95	Avian paramyxovirus 2(APMV-2)	*Metaavulavirus*	APMV-2/Chicken/California/Yucaipa/56	NC_039230.1
96	Avian paramyxovirus 2(APMV-2)	*Metaavulavirus*	NK	HQ896024.1
97	Avian paramyxovirus 2(APMV-2)	*Metaavulavirus*	rAPMV-2-Yuc/YmHA	LC187306.1
98	Avian paramyxovirus 2(APMV-2)	*Metaavulavirus*	APV/chicken/Calfornia/Yucaipa/60	D13977.1
99	Avian paramyxovirus 2(APMV-2)	*Metaavulavirus*	F4	AY129676.1
100	Avian paramyxovirus 2(APMV-2)	*Metaavulavirus*	APMV2/chicken/Jordan/MQA-N-1/2020	ON858805.1
101	Avian paramyxovirus 2(APMV-2)	*Metaavulavirus*	APMV-2/macaw/Taiwan/Q35-SG/2009	MZ802792.1
102	Avian paramyxovirus 2(APMV-2)	*Metaavulavirus*	APMV-2/Emberiza spodocephala/China/Daxinganling/974/2013	KT071757.1
103	Avian paramyxovirus 3(APMV-3)	*Paraavulavirus*	turkey/Wisconsin/68	EU782025.1
104	Avian paramyxovirus 3(APMV-3)	*Paraavulavirus*	APMV3/PKT/Netherland/449/75	EU403085.1
105	Avian paramyxovirus 4(APMV-4)	*Paraavulavirus*	APMV-4/KR/YJ/06	NC_074766.1
106	Avian paramyxovirus 4(APMV-4)	*Paraavulavirus*	APMV-4/Anseriformes/Taiwan/AHRI36/2009	MZ802793.1
107	Avian paramyxovirus 4(APMV-4)	*Paraavulavirus*	APMV4/duck/China/G302/2012	KC439346.1
108	Avian paramyxovirus 4(APMV-4)	*Paraavulavirus*	APMV4/mallard/Belgium/15129/07	JN571485.1
109	Avian paramyxovirus 4(APMV-4)	*Paraavulavirus*	Uria_aalge/Russia/Tyuleniy_Island/115/2015	KU601399.1
110	Avian paramyxovirus 4(APMV-4)	*Paraavulavirus*	APMV-4/european wigeonl/Amur region/Russia/74b/2019	MW959117.1
111	Avian paramyxovirus 4(APMV-4)	*Paraavulavirus*	APMV-4/Egyptian goose/South Africa/N1468/2010	JX133079.1
112	Avian paramyxovirus 4(APMV-4)	*Paraavulavirus*	APMV-4/Mallard/LBM/Korea/019/2012	KY681684.1
113	Avian paramyxovirus 4(APMV-4)	*Paraavulavirus*	APMV-4/Anas sp./Japan/10UO0481/2010	KT732302.1
114	Avian paramyxovirus 4(APMV-4)	*Paraavulavirus*	APMV-4/mallard/Novomychalivka/Ukraine/9-23-12/2010	KT732339.1
115	Avian paramyxovirus 5(APMV-5)	*Metaavulavirus*	APMV-5/budgerigar/Japan/TI/75	LC168750.1
116	Avian paramyxovirus 5(APMV-5)	*Metaavulavirus*	budgerigar/Kunitachi/74	GU206351.1
117	Avian paramyxovirus 6(APMV-6)	*Metaavulavirus*	APMV-6/Anseriformes/Taiwan/AHRI109/2016	MZ802803.1
118	Avian paramyxovirus 6(APMV-6)	*Metaavulavirus*	rAPMV-6-HK199/YmHA	LC187308.1

119	Avian paramyxovirus 6(APMV-6)	*Metaavulavirus*	rAPMV-6-HK199	LC187307.1
120	Avian paramyxovirus 6(APMV-6)	*Metaavulavirus*	APMV-6/duck/HongKong/18/199/77	EU622637.2
121	Avian paramyxovirus 6(APMV-6)	*Metaavulavirus*	APMV-6/duck/Taiwan/Y1/98	AY029299.1
122	Avian paramyxovirus 6(APMV-6)	*Metaavulavirus*	teal/Novosibirsk region/455/2009	KT962980.1
123	Avian paramyxovirus 6(APMV-6)	*Metaavulavirus*	mallard/Jilin/190/2011	JX522537.1
124	Avian paramyxovirus 6(APMV-6)	*Metaavulavirus*	mallard/Jilin/127/2011	KF267717.1
125	Avian paramyxovirus 6(APMV-6)	*Metaavulavirus*	red-crested pochard/Balkhash/5842/2013	KP762799.1
126	Avian paramyxovirus 6(APMV-6)	*Metaavulavirus*	APMV-6/Goose/FarEast/4440/2003	EF569970.1
127	Avian paramyxovirus 7(APMV-7)	*Metaavulavirus*	APMV-7/dove/Tennessee/4/75	FJ231524.1
128	Avian paramyxovirus 8(APMV-8)	*Metaavulavirus*	goose/Delaware/1053/76	NC_039195.1
129	Avian paramyxovirus 8(APMV-8)	*Metaavulavirus*	APMV-8/Little stint/Kazakhstan/14/2013	MF448515.1
130	Avian paramyxovirus 8(APMV-8)	*Metaavulavirus*	APMV-8/WildBird/Anhui/a596/2021	OR271279.1
131	Avian paramyxovirus 8(APMV-8)	*Metaavulavirus*	APMV-8/whooper swan/Kazakhstan/95/2013	MF448514.1
132	Avian paramyxovirus 8(APMV-8)	*Metaavulavirus*	APMV-8/White fronted Goose/Kazakhstan/92/2013	MF448512.1
133	Avian paramyxovirus 8(APMV-8)	*Metaavulavirus*	APMV-8/White fronted Goose/Kazakhstan/62/2013	MF448511.1
134	Avian paramyxovirus 8(APMV-8)	*Metaavulavirus*	APMV-8/pintail/Wakuya/20/78	JX901129.1
135	Avian paramyxovirus 8(APMV-8)	*Metaavulavirus*	APMV-8/Goose/Delaware/1053/76	FJ619036.1
136	Avian paramyxovirus 8(APMV-8)	*Metaavulavirus*	pintail/Wakuya/20/78	FJ215864.1
137	Avian paramyxovirus 8(APMV-8)	*Metaavulavirus*	APMV-8/WildDuck/InnerMongolia/H316/2015	OR271282.1
138	Avian paramyxovirus 9(APMV-9)	*Orthoavulavirus*	APMV-9/Anas sp./Japan/9UO0485/2009	KT867086.1
139	Avian paramyxovirus 9(APMV-9)	*Orthoavulavirus*	duck/New York/22/1978	NC_025390.1
140	Avian paramyxovirus 9(APMV-9)	*Orthoavulavirus*	APMV-9/Mallard/Ningxia/n234/2018	OR271283.1
141	Avian paramyxovirus 9(APMV-9)	*Orthoavulavirus*	PMV-9/mallard/Italy/6226/2008	GU068584.1
142	Avian paramyxovirus 9(APMV-9)	*Orthoavulavirus*	PMV-9/pintail/Italy/493/2004	GU068586.1
143	Avian paramyxovirus 9(APMV-9)	*Orthoavulavirus*	PMV-9/widgeon/Italy/6436/2008	GU068585.1
144	Avian paramyxovirus 9(APMV-9)	*Orthoavulavirus*	PMV-9/mallard/Italy/5709/2007	GU068587.1
145	Avian paramyxovirus 9(APMV-9)	*Orthoavulavirus*	APMV-9/Anas sp./Japan/11OG0839/2011	KT867087.1
146	Avian paramyxovirus 10(APMV-10)	*Metaavulavirus*	penguin/Falkland Islands/324/2007	HM147142.3
147	Avian paramyxovirus 10(APMV-10)	*Metaavulavirus*	rAPMV-10-FI324/YmHA	LC187310.1
148	Avian paramyxovirus 10(APMV-10)	*Metaavulavirus*	rAPMV-10-FI324	LC187309.1
149	Avian paramyxovirus 10(APMV-10)	*Metaavulavirus*	APMV-10/penguin/Falkland Islands/323/2007	HM755886.2
150	Avian paramyxovirus 10(APMV-10)	*Metaavulavirus*	APMV-10/Penguin/Falkland Islands/539/2007	HM755888.2
151	Avian paramyxovirus 10(APMV-10)	*Metaavulavirus*	APMV-10/Penguin/Falkland Islands/437/2007	HM755887.2
152	Avian paramyxovirus 11(AMPV-11)	*Metaavulavirus*	common_snipe/France/100212/2010	JQ886184.1
153	Avian paramyxovirus 12(AMPV-12)	*Orthoavulavirus*	Wigeon/Italy/3920_1/2005	KC333050.1
154	Avian paramyxovirus 12(AMPV-12)	*Orthoavulavirus*	APMV-12/Anseriformes/Taiwan/AHRI101/2015	MZ802804.1

155	Avian paramyxovirus 12(AMPV-12)	*Orthoavulavirus*	APMV-12/Anseriformes/Taiwan/AHRI143/2019	MZ802805.1
156	Avian paramyxovirus 13(APMV-13)	*Orthoavulavirus*	APMV-13/white fronted goose/Northern Kazakhstan/5751/2013	KU646513.1
157	Avian paramyxovirus 13(APMV-13)	*Orthoavulavirus*	white-fronted goose/Ukraine/Prymorske/71-15-02/2013	OQ286221.1
158	Avian paramyxovirus 13(APMV-13)	*Orthoavulavirus*	AOAV-13/Greater white-fronted goose/South Korea/E20-158-3/2020	OK513542.1
159	Avian paramyxovirus 13(APMV-13)	*Orthoavulavirus*	AOAV-13/wild goose/China/Hubei/V93-1/2015	MN150295.1
160	Avian paramyxovirus 13(APMV-13)	*Orthoavulavirus*	white-fronted goose/Ukraine/Askania-Nova/48-15-02/2011	KX119151.2
161	Avian paramyxovirus 13(APMV-13)	*Orthoavulavirus*	goose/Shimane/67/2000	NC_030231.1
162	Avian paramyxovirus 13(APMV-13)	*Orthoavulavirus*	APMV-13/WildBird/Heilongjiang/x170/2018	OR271285.1
163	Avian paramyxovirus 13(APMV-13)	*Orthoavulavirus*	APMV-13/wildbird/Anhui/a435/2019	OR271284.1
164	Avian paramyxovirus 13(APMV-13)	*Orthoavulavirus*	Greater white-fronted goose/Korea/29-9/2017	MW492545
165	Avian paramyxovirus 14(APMV-14)	*Metaavulavirus*	APMV14/duck/Japan/11OG0352/2011	NC_039015.1
166	Avian paramyxovirus 14(APMV-14)	*Metaavulavirus*	chicken/Fujian/2160/2020	MZ351191.1
167	Avian paramyxovirus 14(APMV-14)	*Metaavulavirus*	chicken/Fujian/1013/2022	OP094833.1
168	Avian paramyxovirus 14(APMV-14)	*Metaavulavirus*	duck/Jiangxi/1232/2022	OP094832.1
169	Avian paramyxovirus 15(APMV-15)	*Metaavulavirus*	APMV-15/calidris_fuscicollis/Brazil/RS-1177/2012	NC_034968.1
170	Avian paramyxovirus 16(APMV-16)	*Orthoavulavirus*	APMV-15/WB/Kr/UPO216/2014	NC_039016.1
171	Avian paramyxovirus 16(APMV-16)	*Orthoavulavirus*	APMV-16/BeanGoose/Hubei/B926/2016	OR271292.1
172	Avian paramyxovirus 16(APMV-16)	*Orthoavulavirus*	APMV-16/WildDuck/Heilongjiang/x1061/2018	OR271290.1
173	Avian paramyxovirus 16(APMV-16)	*Orthoavulavirus*	APMV-16/WildBird/Anhui/a454/2019	OR271288.1
174	Avian paramyxovirus 16(APMV-16)	*Orthoavulavirus*	APMV-16/BeanGoose/Hubei/B931/2016	OR271293.1
175	Avian paramyxovirus 16(APMV-16)	*Orthoavulavirus*	APMV-16/WildBird/Anhui/a482/2019	OR271289.1
176	Avian paramyxovirus 16(APMV-16)	*Orthoavulavirus*	APMV-16/BeanGoose/Hubei/B281/2016	OR271294.1
177	Avian paramyxovirus 16(APMV-16)	*Orthoavulavirus*	APMV-16/white fronted goose/Central Kazakhstan/1791/2006	MH423285.2
178	Avian paramyxovirus 16(APMV-16)	*Orthoavulavirus*	APMV-16/GTeal/Heilongjiang/x811/2018	OR271296.1
179	Avian paramyxovirus 16(APMV-16)	*Orthoavulavirus*	AOAV-16/emperor goose/Alaska/AK19-296/2019	MW161159.1
180	Antarctic penguin virus A(APMV-17)	*Orthoavulavirus*	Adelie penguin 14	MT025071.1
181	Antarctic penguin virus A(APMV-17)	*Orthoavulavirus*	A	KY452442.1
182	Antarctic penguin virus A(APMV-17)	*Orthoavulavirus*	Antarctic12	MT025076.1

183	Antarctic penguin virus B(APMV-18)	*Orthoavulavirus*	B	NC_039018.1
184	Antarctic penguin virus B(APMV-18)	*Orthoavulavirus*	AAvV-18/Adelie penguin/Antarctica/132/2013	MK167230.1
185	Antarctic penguin virus B(APMV-18)	*Orthoavulavirus*	AAvV-18/Adelie penguin/Antarctica/98/2013	MK167229.1
186	Antarctic penguin virus C(APMV-19)	*Orthoavulavirus*	C	NC_039019.1
187	Antarctic penguin virus C(APMV-19)	*Orthoavulavirus*	AAvV-19/Adelie penguin/Antarctica/167/2013	MK167232.1
188	Antarctic penguin virus C(APMV-19)	*Orthoavulavirus*	AAvV-19/Adelie penguin/Antarctica/96/2013	MK167231.1
189	Antarctic penguin virus C(APMV-19)	*Orthoavulavirus*	AAvV-19/Chinstrap penguin/Antarctica/661/2013	MK167233.1
190	Avian paramyxovirus 20(APMV-20)	*Metaavulavirus*	APMV-20/gull/Kazakhstan/5976/2014	NC_040796.1
191	Avian paramyxovirus 20(APMV-20)	*Metaavulavirus*	AAvV-20/great black headed gull/Atyrau/5541/2013	MH844488.1
192	Avian paramyxovirus 20(APMV-20)	*Metaavulavirus*	AAvV-20/black headed gull/Balkhash/5844/2013	MH844489.1
193	Avian paramyxovirus 21(APMV-21)	*Orthoavulavirus*	APMV-21/Anseriformes/Taiwan/AHRI141/2019	MZ802807.1
194	Avian paramyxovirus 21(APMV-21)	*Orthoavulavirus*	Cheonsu1510	NC_076003.1
195	Avian paramyxovirus 21(APMV-21)	*Orthoavulavirus*	APMV-21/Anseriformes/Taiwan/AHRI83/2013	MZ802806.1
196	Avian paramyxovirus 21(APMV-21)	*Orthoavulavirus*	APMV/dove/Taiwan/AHRI104/2016	MK677432.1
197	Avian paramyxovirus 21(APMV-21)	*Orthoavulavirus*	APMV/pigeon/Taiwan/AHRI128/2017	MK677433.1
198	Avian paramyxovirus 21(APMV-21)	*Orthoavulavirus*	APMV/dove/Taiwan/AHRI48/2010	MK677437.1
199	Avian paramyxovirus 21(APMV-21)	*Orthoavulavirus*	APMV/dove/Taiwan/AHRI58/2010	MK677443.1
200	Avian paramyxovirus 21(APMV-21)	*Orthoavulavirus*	APMV/dove/Taiwan/AHRI53/2010	MK677440.1
201	Avian paramyxovirus 21(APMV-21)	*Orthoavulavirus*	APMV/dove/Taiwan/AHRI33/2009	NC_076397.1
202	Avian paramyxovirus 21(APMV-21)	*Orthoavulavirus*	APMV/dove/Taiwan/AHRI55/2010	MK677442.1
203	Avian paramyxovirus 22(APMV-22)	*Metaavulavirus*	APMV-22/dove/Taiwan/AHRI140/2019	MZ802809.1
204	Avian paramyxovirus 22(APMV-22)	*Metaavulavirus*	APMV-22/pigeon/Taiwan/Q97-NL/2015	MZ802808.1
